# Dentist Mothers’ Attitudes, Challenges and Facilitators in the Oral Health Promotion of Their Young Children

**DOI:** 10.3390/children11010059

**Published:** 2023-12-31

**Authors:** Damla Akşit-Bıçak

**Affiliations:** Department of Pediatric Dentistry, Faculty of Dentistry, Final International University, Mersin 10, Nicosia 99010, Turkey; damla.bicak@final.edu.tr

**Keywords:** barriers, behavior management, children, dentist, early childhood caries, facilitators, mother, night feeding, nutrition, oral health, parental supervised brushing, toothbrushing

## Abstract

Twice daily parental supervised brushing (PSB) is recommended for the effective oral hygiene of children with toothpaste including fluoride. This cross-sectional study aimed to identify dentist mothers’ attitudes, challenges, and facilitators in the oral health promotion of their young children. An online questionnaire was prepared and distributed to dentists via e-mail with a link to the form. Of all the mothers, 46.50% started brushing their children’s teeth after the eruption of the first primary teeth. Nearly 50% of the mothers thought that they should brush their children’s teeth until at least the age of 7–8 years old. In terms of the main barriers to toothbrushing, 52.9% said children could fall asleep at home or in the car, while 27.5% of all mothers reported being tired as the main barrier to toothbrushing. Although dentist mothers have sufficient knowledge to promote oral and dental health, they do not always have ideal attitudes and behaviors and they may encounter various barriers regarding themselves and their children in practical applications. Providing oral care for young children goes beyond ‘knowledge’ and is sometimes ‘challenging’; however, it could be ‘possible’ by developing and implementing the most appropriate solution strategies suitable for each unique family and child.

## 1. Introduction

Oral health is a crucial part of overall health and has an influence on the quality of life of children by ensuring mental, physical, and social well-being. In cases where good oral health cannot be achieved, an important public health problem, namely ‘early childhood caries’ (ECC), can be observed on the primary dentition of young children [1,2]. Thus, soft and hard tissues of the oral cavity are negatively affected and children can suffer from pain and discomfort, as well as speaking, eating, growing, and socializing problems [3,4,5]. Therefore, primary prevention of ECC has importance because it is easier to prevent the formation of caries than perform treatment that generally needs extensive restorative and surgical treatments of primary teeth, which can be psychologically and financially challenging for children and their families [3,6,7]. Since infants and toddlers are unable to care for themselves and are dependent on their parents, parents, and especially mothers, are primarily responsible for sustaining the oral health of young children as primary caregivers [8,9,10].

Children’s dental caries experiences in the period of primary dentition can cause the same experiences in permanent dentition. For this reason, preventing caries formation in early childhood is very important for maintaining oral health throughout life [11]. In addition, it has been stated that, in spite of adherence to preventive measures, the development of dental caries indicates that personal care at home has a greater influence than professional applications in the development of dental caries [12]. Thus, twice daily parental supervised brushing (PSB) is recommended for the effective oral hygiene of children with toothpaste including fluoride, which should begin as soon as the first primary tooth erupts and continue until at least 7 years of age [13,14]. During PSB, caregivers brush children’s teeth and children are not refusing this care. Individual characteristics of the parent and child and, also, the interpersonal characteristics of their relationship both effect PSB [13,15]. There are various different barriers to PSB, which were structured onto the theoretical domains framework (TDF) including social and environmental factors, knowledge, attitudes, beliefs about capabilities (self-efficacy), behavior management, and the feature of the behaviors as main barriers. Parents who have experienced difficulties with PSB have reported that their parenting skills were not sufficient to manage their children’s reluctant behavior when they did not want to brush their teeth [13,16]. The main barrier to twice daily toothbrushing that most parents frequently encounter is child refusal. Thus, even when parents are willing and knowledgeable about the importance of oral health care, they face difficulties with establishing a routine. It is demonstrated that child compliance and the behavior management skills of parents could be related with improved oral health [17,18].

In order to ensure children’s oral health, parents need to have good oral health knowledge and attitudes and they must put them into practice [19]. However, good knowledge and attitudes supporting oral health do not inevitably produce good practices [20]. Despite the fact that parents have sufficient knowledge about maintaining the oral health of children, the resistance of children to toothbrushing and the lack of personal skills of their families in overcoming these difficulties are the main barriers to maintaining appropriate oral hygiene of children [21,22]. Thus, knowledge and practice should act together to improve oral health care of children. 

In order to clarify the personal skills and barriers of parents involved in toothbrushing and behavior regulation of young children, this study was conducted on dentist mothers who understand the importance of primary teeth and have sufficient knowledge related to oral health. So, the purpose of this study was to identify dentist mothers’ attitudes, challenges, and facilitators in the oral health promotion of their young children. This study is based on the hypothesis that, although dentist mothers have sufficient knowledge about oral and dental health, they may encounter various barriers regarding themselves and their children in practical applications and that they cannot always have ideal attitudes and behaviors.

## 2. Materials and Methods

### 2.1. Ethical Considerations

Ethical approval for this study was obtained from the Near East University Scientific Research Ethics Committee (application number NEU/2019/69-834) on 30 May 2019.

### 2.2. Study Participants

Within the scope of this cross-sectional study, it was planned to reach female dentists with one or more children up to 10 years old and who were registered with the Dental Chambers of the Turkish Dental Association. G∗ Power (Version 3.1.9.4) for Mac software was used for sample size calculation. Based on similar studies [3,4,5,8,9,12,13,16,17,18,19,20] in the literature and as a result of statistical evaluation with 80% statistical power and 5% margin of error, a sample size of 150 was found to be representative of the total dentist mother population.

An online questionnaire made available on the Google Forms platform (Google Inc., Menlo Park, CA, USA) in Turkish was prepared after conducting a review of the recent literature [16,17,18,23,24], and necessary corrections were made after piloting on 30 colleagues. Participation in the study was on a voluntary basis. Inclusion criteria (being female dentists with one or more children up to 10 years old), aim of the study, and consent to participate were included as a covering letter and participants who approved were able to proceed to the survey questions. The questionnaire was distributed to the registered dentists via the e-mail of the Turkish Dental Association with a link to the form in July 2019 and responses were accepted until the end of February 2020. If the participants had more than one child, they were asked to answer the questionnaire on the basis of their youngest child. No identifying information was obtained from the study participants. No reminder was sent.

### 2.3. Survey Tool and Data Collection

The questionnaire consisted of 54 questions in total, which included open-ended, closed-ended, Likert scale, matrix, dropdown, and multiple-answer multiple-choice questions, and was divided into three parts. The first and second parts of the survey included demographic data such as age, gender, father’s education level, and dental specialties of the mothers. In addition, the children’s nutrient consumption habits, night feeding habits, oral health habits, and oral bad habits were assessed. The third part included questions regarding parental supervised toothbrushing. This part of the survey also included questions regarding the challenges/barriers faced by dentist mothers when promoting the oral health of their children and facilitators used while ensuring their children’s oral hygiene in the past or present. Furthermore, by using open-ended questions, the participants had the opportunity to give more feedback through a text box. The average time required to complete the questionnaire is 15–20 min.

### 2.4. Statistical Analysis

In this study, statistical analyses were performed with the Number Cruncher Statistical System (NCSS) 2007 Statistical Software (Kaysville, UT, USA) package program. Descriptive statistical methods (mean, standard deviation, frequency, and percentage distributions) as well as chi-square test were used in the evaluation of the data. The results were evaluated at the significance level of *p* < 0.05.

## 3. Results

A total of 162 mothers participated in the study. Incompletely filled questionnaire forms were excluded and 156 questionnaire forms were selected for further evaluation. Thus, the final study sample was comprised of 156 children and their mothers, which included 79 girls with a mean age of 4.28 ± 2.33 and 77 boys with a mean age of 4.32 ± 2.31. The mean age of all the children was 4.30 ± 2.31 and ranged from 0 to 10 years. The mean age of the mothers was 36.12 ± 4.74 years, ranging from 25 to 54 years (Table 1).

Approximately 27% of the fathers were general dentist and dental specialists, 30.57% had a Master’s–PhD degree, 38.85% had a university education, and 3.18% had a high school education. Among the mothers, 51.28% were general dentists, 21.79% were pediatric dentistry specialists, and other dentists had different specialties, as shown in Table 2.

### 3.1. Nutritional Habits

Analysis of the questionnaire about the children’s nutritional habits revealed that 58.71% of all children never consumed candy or sweet foods, 66.67% never consumed snacks and potato chips, and 87.10% never consumed carbonated beverages. A total of 48 percent of children consumed chocolate and cookies several times a week, 40.76% consumed milk and milk products multiple times during the day, and 40% consumed fresh vegetables and fruits multiple times during the day (Table 3). It was reported that 59.35 percent of children consumed cariogenic foods (sugars, biscuits, and chocolate) in school and in the family environment beyond their mother’s control.

### 3.2. Night Feeding Habits

In response to the question asked to mothers regarding night feeding habits, it was found that 13.38% of all children still continued night feeding and 29.93% of the children stopped night feeding by the age of 1 (Table 4).

Nearly 61% of children never consumed formula milk during night feeding. On the other hand, 43.31% of all children always consumed breast milk at night, while 29.30% of all children generally consumed breast milk at night. A majority of mothers (74.03%) could not clean their children’s teeth after night feeding, 12.99% of all mothers used wet gauze, and 5.84% used a finger brush without paste. The reason for not being able to clean their children’s teeth after night feeding was they did not want to wake their baby up in most cases (69.86%) (Table 4). The distribution of answers to all questions related with the night feeding habits of children is presented in Table 4.

### 3.3. Oral Health Status/Habits

Of all the mothers questioned, 46.50% of mothers started brushing their children’s teeth after the eruption of the first primary teeth, while 28.66% of mothers started cleaning tooth crests before the eruption of primary teeth and kept brushing after the eruption of the first primary teeth (Table 5). The distribution of answers to all questions related with the oral health status/habits of children is presented in Table 5.

Finger brush and toothbrush without paste usage was found to be statistically significantly higher, while toothbrushing with fluoride-free and fluoridated paste usage were found to be statistically lower in children between 0 and 2 years old (*p* < 0.05). Fluoride-including toothpaste usage was found to be statistically significantly lower in children between 0 and 2 years old (*p* = 0.001). No statistically significant difference was detected between ages in terms of the amount of toothpaste used (*p* = 0.190) (Table 6). It was found that 28.21% of all mothers applied professional fluoride varnish/gel to their children and 71.79% did not (Table 5). Among the fluoride non-applied group, the number of children between 0 and 2 years old was found to be statistically higher than those of other ages (*p* = 0.001) (Table 6). Most of the mothers were satisfied with the frequency at which their children brushed their teeth, as 21.94% percent of mothers were always and 50.32% percent of mothers were generally satisfied. Most of the mothers (96.82%) reported that their children regarded them as an example for their own toothbrushing habit and most of the mothers (95.54%) thought that they were an example to their children in terms of toothbrushing habits (Table 5). When answering the open-ended question, one mother indicated that ‘You are the best role model for the child. If your hygiene is good, your child’s will also be good’.

### 3.4. Bad Oral Habits

A majority of children (45.51%) never used pacifiers, whereas 17.95% used them up to 1 to 2 years of age, 14.10% used them up to 2.5 years of age, and 5.77% were still using a pacifier, which largely comprised children between 0 and 2 years old (*p* = 0.0001) (Table 6). Among these children, 56.13% of them did not need a pacifier during night sleep, while 29.03% of them needed a pacifier to sleep at night but the mothers took it out of the child’s mouth when they were sleeping, and 14,84% of them needed a pacifier throughout the night (Table 7). The distribution of answers to all questions related with oral bad habits of children is presented in Table 7.

### 3.5. Parental Supervised Toothbrushing and Behavior of Children during Toothbrushing

Half of the mothers thought that they should brush their children’s teeth until at least 7–8 years old. Overall, 34 percent of all mothers brushed their children’s teeth, whereas 34.84% of all mothers supervised their children during toothbrushing and often performed the brushing themselves (Table 8). The percentage of mothers who brushed their children’s teeth was found to be statistically significantly higher in the 0–2 age group than other ages (*p* = 0.0001). The percentage of mothers who supervised their children during toothbrushing and often performed the brushing themselves was found to be statistically significantly higher in the 2–6 age group than other ages (*p* = 0.022). The percentage of mothers who supervised their children during toothbrushing and sometimes performed the brushing was found to be statistically significantly higher in the >6 age group than other ages (*p* = 0.008) (Table 6). 

A total of 62 percent of mothers declared that it was very easy to maintain their children’s oral health care in their daily routine, while 13.46% stated that it was very easy, 20.51% said it was hard, and 3.85% percent declared that it was very hard (Table 8). The frequency of toothbrushing three times a week was found to be statistically significantly higher in the hard and very hard group than the very easy and easy groups (*p* = 0.001).

Of all the mothers, 55.77% of them stated that they always had the greatest responsibility for ensuring their children’s oral hygiene. It was found that 20.65% of all children were co-operative, 56.77% participated, 15.48% were resistant, 2.58% were uncooperative, and 4.52% exhibited independent behavior during toothbrushing (Table 8).

In total, 70 percent of all mothers never applied physical restraint to their children if they showed resistance to toothbrushing, whereas 22.08 percent sometimes applied such restraint. Fifty-five percent mothers forced their children to perform toothbrushing. Thirty-two percent of mothers declared that they experienced the most difficulty in brushing their children’s teeth when they were between 1 and 2 years of age. Ninety-nine percent of mothers thought that parents’ ability to manage their children’s behavior (parental skills) and interpersonal communication skills are important in protecting children’s oral and dental health (Table 8).

### 3.6. Barriers and Facilitators in Oral Health Promotion of Young Children

**Figure 1:**
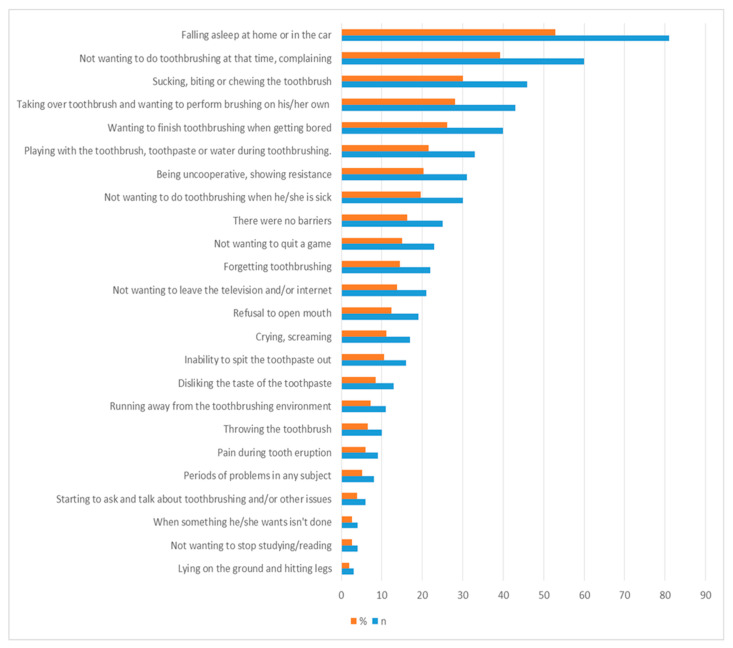
Distribution of barriers related with children while ensuring parental supervised toothbrushing (n: number, %: percentage. Participants could choose more than one option).

Figure 1 provides the main barriers/challenges related to children’s toothbrushing in the past or present. A total of 52.9 percent said their children could fall asleep at home or in the car, 39.2% said their children complained that they did not want to brush their teeth at that time, and 30.1% stated that their children suck, bite, or chew the toothbrush during toothbrushing. All barriers related to children are given in Figure 1.

**Figure 2:**
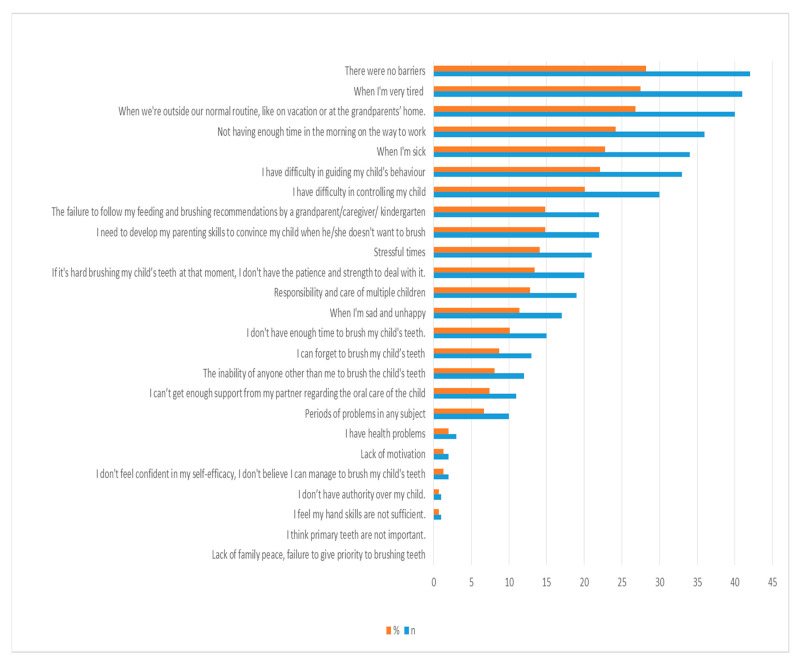
Distribution of barriers related with mothers while ensuring parental supervised toothbrushing (n: number, %: percentage. Participants could choose more than one option).

All barriers related to mothers while ensuring their children’s oral hygiene in the past or present are shown in Figure 2. Of all mothers, 27.5% of them reported being tired, 26.8% of them reported being outside their normal routine, such as on vacation or at the grandparents’ home, and 24.2% of them reported not having enough time in the morning while preparing for work.

**Figure 3:**
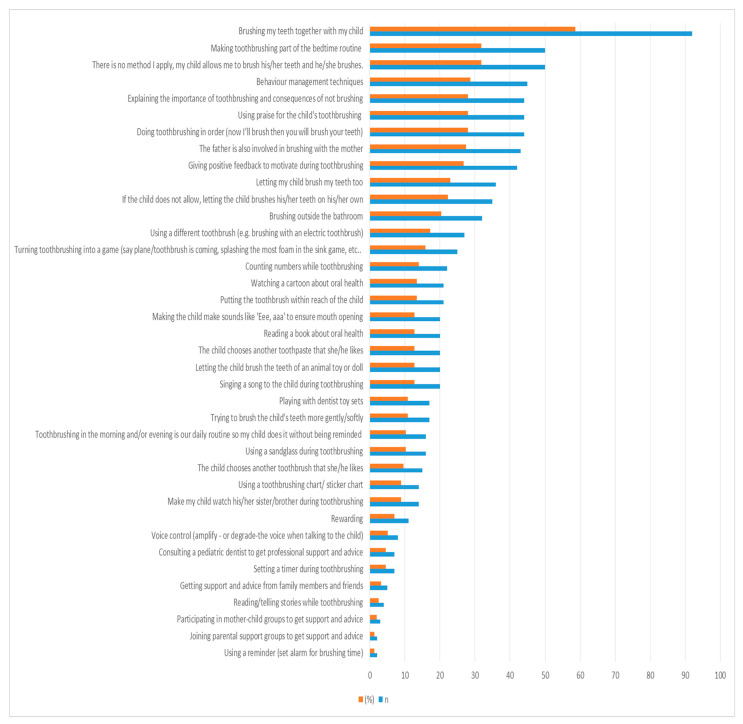
Distribution of facilitators used by mothers while ensuring parental supervised toothbrushing (n: number, %: percentage. Participants could choose more than one option).

In terms of the main facilitators, 58% of all mothers brushed their teeth together with their children and 31.8% of all mothers included toothbrushing in the bedtime routine. All facilitators used by mothers while ensuring their children’s oral hygiene are shown in Figure 3. By answering the open-ended question, the participants’ expressed their ideas on this topic: ‘Especially for the 5–8 year olds, playing with the applications on tablets and phones under parental control also helps very much during brushing’. Furthermore, one mother stated that ‘brushing the child’s teeth while the father is tickling helps us during brushing’.

## 4. Discussion

Habits related with oral health are acquired during early childhood; thus, the oral health of children is mainly affected by the knowledge of the parents related to oral health [25]. As indicated clearly in the previous literature [25,26,27,28], oral health knowledge is mandatory to implement good oral health behaviors. Furthermore, reflection of the knowledge in attitudes and practices constitutes a second and important step on the path to achieving good oral health. Because of some barriers related with parents and children, it is sometimes very hard to put knowledge into practice. Mothers are generally recognized as principal caregivers [29]. Thus, this study was conducted on dentist mothers with a high level of oral health knowledge for a number of reasons: firstly, in order to examine their oral health practices; secondly, to clarify the most significant barriers related to children and mothers; and, lastly, to examine facilitators in order to overcome these barriers in the oral health promotion of young children.

A study by Wigen and Wang [8] showed that children of parents with low education levels were 12 times more likely to develop cavities before the age of 5 than other children. Another study [30] reported that, considering the educational background, illiterate mothers have insufficient knowledge, attitudes, and bad practices regarding the oral hygiene of their children. In most of the previous literature [26,27,30,31,32,33,34,35,36], it has been shown that highly educated parents have significantly better knowledge, attitudes, and practices towards the oral health care of their children. The education levels of the parents participating in the current study were high and they mostly exhibited good attitudes and practices while promoting their young children’s oral health. Oral health knowledge is the first and most important step in oral health promotion of young children. It is obvious that, without knowledge, there will be no good attitudes and practices towards oral health care. 

Dietary behaviors are also critical in the progression of dental caries, and it is clear that sugar consumption in children is associated with caries development [8]. In the literature, a number of studies [37,38,39,40,41,42] have examined the parental knowledge of dietary habits related to the formation of dental caries. The results of these studies [37,38,39,40,41,42] supported the assumption that the vast majority of parents have good knowledge of the importance of dietary habits and the role of sweet and sticky foods in the development of dental caries. Practical reflection of the knowledge into nutrient consumption habits was shown in the current study in order to provide good oral health. Previous studies [5,24,31,36,40,43,44,45,46] have reported higher cariogenic food consumption among children compared to the current study. In some studies [24,31], it is stated that the high rate of cariogenic food consumption in children of mothers with high educational status is an issue that should be evaluated in terms of the prevention of early childhood caries. This situation highlights the need for oral and dental health education to be given to all mothers regardless of their education and socioeconomic status. Furthermore, in this study, mothers reported that cariogenic feeding occurred when the children were away from their mothers in the school and family environments as in line with previous studies [43,47]. Children’s access to cariogenic foods should be restricted in the school environment and extended family members should pay attention to the sensitivity of parents regarding the consumption of such foods.

Primary prevention of ECC includes preventing bottle feeding, including milk or any other sugary drinks, during nights and avoiding breastfeeding after 12 months [48]. In some studies [4,39,42,49], the knowledge and awareness of the parents about the effect of night feeding on the formation of dental caries were found to be satisfactory. On the other hand, others [38,40,41,45] have reported that parents did not have sufficient knowledge about the consequences of night feeding in young children. In addition to the knowledge and awareness of the parents, night feeding practices are an important issue that must be discussed. The results of the current study reveal better night feeding practices than those found in previous studies [26,31,50]. 

The majority of mothers who participated in this study could not clean their children’s teeth after night feeding. The reasons for not being able to clean their children’s teeth after night feeding in most cases were to not wake the baby up, followed by being tired and other reasons. Therefore, according to the results of the present study, dentist mothers were knowledgeable about the impact of night feeding on oral health and they were in an effort to breastfeed their children. The reasons for the prolongation of night feeding after 1 year of age in this study might be because of breastfeeding habits, nearly half of the babies never used pacifiers, and mothers thought that they could perform oral cleaning when their children woke up in the morning and, thus, this would not allow plaque build-up and they did not use sugar-containing foods anyway.

Dental caries is a transmissible infectious disease in which Streptococcus mutans is generally considered to be the main etiological agent. Sharing food and utensils with children, breastfeeding, and sleeping together, which includes close contact with the child, can facilitate the transfer of saliva from adults to children, causing early colonization of streptococcus mutans [4,26]. In previous studies [31,38,39,40,42,45,50,51,52], the knowledge and awareness of parents about this fact were found to be insufficient. However, in other studies [53,54], it was found to be high. The findings of this study were found to be below expectations fortunately; a majority of the mothers (55.77%) never shared utensils with their children. 

White spot lesions, as in opaque and milky-white color, are the early signs of dental caries and, if not treated, restorative treatment is mandatory [55]. Nearly 12% of all children had white spot lesions and 16.67% had cavitated carious lesions in this study. In order to prevent caries formation, oral hygiene practices should start in the first year of infancy before the first tooth eruption to protect young children from dental caries [4,28]. Tooth buds of babies can be cleaned with gauze or a muslin cloth from birth [29]. In the study of Elison et al. [29], starting tooth brushing as early as possible was accepted by almost all mothers. Kamil et al. [42] stated that approximately 44% of the participants reported that it is important to start cleaning the mouth of the infants immediately after birth. In some studies [4,32,50,53,56], it was found that most of the participants started brushing their babies’ teeth when the first deciduous teeth erupted. On the other hand, in other studies [27,34,49,57,58], most of the parents thought that their children should brush their teeth later on and after all primary teeth have erupted. In this study, most of the mothers said they started brushing their children’s teeth after the eruption of the first primary teeth, in accordance with previous research [4,32,50,53,56,59]. In this study, nearly 75% of mothers said they started toothbrushing immediately after birth and at least after the eruption of the first primary teeth. Furthermore, previous research [27,32,34,60] suggests that caregivers may not have clear information about whether fluoride toothpaste is suitable for children under three years of age. In this study, half of the mothers reported using fluoride-free toothpaste and only 33.78% of children use fluoridated toothpaste. These findings were found to be low when compared with Elison et al.’s study [29] in which all mothers reported they used fluoridated toothpaste. In a recent study, higher parental education level was found to be associated with the use of non-fluoridated dentifrice [61].

In the study of Akshehri and Nasim [4], 20.98% of the parents reported that they provided oral hygiene only with water, 27.87% with wet cotton, 28.20% with a toothbrush, and 10.16% with toothpaste. In another study [38], it was reported that 32% of the parents used a toothbrush and toothpaste and 13.7% used finger brushes and toothpaste/powder in order to clean their children’s teeth. Sultan et al. [52] reported that 81% of mothers preferred a toothbrush and toothpaste. In this study, it was determined that a toothbrush or finger brush without toothpaste was generally used among children aged 0–2 years. If toothpaste was used for children in this age group, fluoride-free paste was preferred. In our opinion, fluoride paste is not preferred by mothers because babies in this age group have a risk of swallowing paste, which can cause dental fluorosis. A recent review reported that children who start brushing with fluoride toothpaste after 24 months have a reduced risk of dental fluorosis compared to children who use fluoride toothpaste before 24 months of age [62]. Brushing the child’s teeth twice a day with toothpaste containing at least 1000 ppm fluoride is very effective in reducing dental caries. The amount of paste appropriate for the age of the child should be used. This amount should be the size of a smear for children under 3 years old and a pea-grain-sized amount for children aged 3 to 6 years old [59]. This knowledge is important for parents to avoid excess toothpaste ingestion [49]. In this study, more than half of mothers reported that they used a smear layer of toothpaste and 43.8% used a pea-sized amount of toothpaste, which is line with previous studies [40,49,50]. The results of the study of Wright et al. [62] showed that usage of fluoridated toothpaste among children below 6 years of age is effective in caries control. However, researchers have reported that swallowing pea-sized amounts or larger can cause mild fluorosis. Furthermore, only 28% of all mothers applied professional fluoride varnish/gel to their children in the current study. Among the non-fluoride-applied group, the number of children between 0 and 2 years old was found to be statistically higher than those from other age groups. This may be due to the fact that the children of dentist mothers do not consume a cariogenic diet and the mothers regularly brush their babies’ teeth and that the mothers consider them to be in the low-caries-risk group as they are constantly under their control. Furthermore, dentist mothers understand the importance of mechanical plaque removal and may use other products including other remineralization agents rather than fluoride.

When toothbrushing frequency is examined, 49% of children brushed at least two times a day and nearly 6.5% brushed after each feeding. Also, 38.71% of children brushed their teeth once per day, only in the evening before bedtime in the current study. In other studies [28,31,32,34,35,40,41,52,60,63], toothbrushing was performed less than two times among most of the children. However, other researches [24,27,29,33,46,57,64] has reported that toothbrushing was conducted at least two times a day among most of the children. Furthermore, tongue cleaning with a toothbrush or tongue cleaner has been reported to result in a significant reduction in streptococcus mutans count and plaque levels. Winnier et al. [27] reported that 82.8% of the parents in their study regularly cleaned their child’s tongue. Similarly, in the current study, nearly 67% of the children’s tongue surface was brushed at least sometimes. 

Parents’ behaviors and attitudes towards oral health can greatly affect their children’s oral-health-related behaviors [8]. For example, if the parents brush their teeth twice a day, their children will be positively affected by brushing their teeth twice a day [28]. Previous research [65,66,67,68,69] has reported a direct relationship between children’s and mothers’ toothbrushing habits. During childhood, parents, especially the mother, play an important role in shaping the oral health behaviors of the child. Therefore, it can be said that consistent oral health behavior habits start to be acquired at home and parents should be informed that their own dental health habits affect their children’s oral health and, therefore, their quality of life [68]. In accordance with the literature, in this study, nearly all of the mothers reported that their children accepted them as an example for their own toothbrushing habit and think that they are an example to their children about toothbrushing habits. In line with the literature, in the current study, it was detected that the mothers have good oral health and their children also have good oral health. Furthermore, the dental caries prevalence of dentist mothers’ children was low, as expected. It was clearly observed that the oral hygiene of the children was in a good condition as a result of their appropriate diet, good brushing habits, and constant oral check-ups by their dentist mothers.

It has been reported that supervised toothbrushing programs in schools improve the oral health of children [70]. Although parents play the most important role in protecting their children’s oral health, children spend most of the day at school. For this reason, school teachers should also be included in the oral health education of children. Teachers can explain the importance of consuming less sugary foods and drinks to children and inform them about this issue [68]. Parents, teachers, and dentists are mainly responsible for oral health good practices [58]. In this study, it was found that only a limited number of children could brush their teeth at school, in line with the findings of Togoo et al. [54]. So, most of the mothers in this study supported the development of toothbrushing protocol in order to promote toothbrushing at school under the supervision of teachers. 

When the bad oral habits of the participating mothers’ children were examined, it was detected that a vast majority of children never used pacifiers and more than half did not need a pacifier during night sleep. However, it has been reported that pacifier use can decrease sudden infant death syndrome incidence and can be recommended to the mothers of healthy term infants when babies are nearly 3 to 4 weeks of age during infant nap or sleep time after breastfeeding [71]. It was determined that the majority of the children participating in the current study did not have bad oral habits such as finger sucking, mouth breathing, bruxism, nail eating, lip biting, tongue thrusting, or lip sucking habits. Only 10.26% of children developed malocclusion due to bad oral habits. It is clear that mothers have the knowledge about the impact of oral bad habits on the developing dentition and jaws and they can apply their knowledge in practice. In previous studies, Al Jameel et al. [39] reported that more than 86% of mothers, Jain et al. [34] reported that 46.2% of parents, Sehrawat et al. [32] reported that 33.1% of mothers, and Monahar and Mani [28] reported that only 23% of the parents were educated about the consequences of oral habits such as tongue thrusting, mouth breathing, and thumb sucking on children’s developing dentition. 

Another important aspect in the oral health promotion of young children is parental supervised toothbrushing. Children under the age of 7 should be closely supervised when brushing their teeth for several reasons. First, children only acquire the necessary cognitive and motor skills to brush their teeth in mid-childhood. The second is that young children’s mouths are very sensitive. Therefore, if children are left unattended while brushing their teeth, they can potentially cause damage to their mouths with their toothbrushes [29]. Brushing teeth can be taught to children in the same way as any other skill. However, in order to ensure that all areas of the mouth are cleaned each time, it should be supervised closely on a regular basis [56]. As children grow up, they acquire both fine and gross motor skills. Ogasawara et al. [72] explained that the skill of learning to brush teeth and the establishment of the toothbrushing habit are two different things. They also suggested that children should be given constant guidance on brushing until their toothbrushing habits are embedded in their daily lives. Unfortunately, good oral hygiene in children is often difficult due to lack of motivation and poor dexterity [72,73]. The AAPD guidelines also state that parents should supervise their children’s brushing at least 8 years. The age of 7–8 is the point at which fine motor skills develop [27]. In line with the literature, in this study, more than half of the mothers thought that they should brush their children’s teeth until at least 7–8 years old. In contrast, in the study of Gussy et al. [50], 52% of the parents reported that, around the age of four years, their children were capable of brushing their own teeth. Unfortunately, those children who brushed their teeth alone had almost twice the amount of caries than children who were helped by parents [49]. Almost all mothers who participated in our study at least supervised and 92.9% reported brushing the teeth of their children by themselves. In line with our finding, in previous studies [5,34,46,47,49,55,73,74], most of the parents were aware and adhered to supervised toothbrushing. However, the outcomes of some reports [19,28,30,35,38,44,57,64,75] were not in accordance with our findings and showed a lack of knowledge and practice regarding parental supervised toothbrushing among most of the participant parents. Winnier et al. [27] noted that 48.2% of parents agreed that the supervision of brushing is required until the age 7–8 years; however, about 67.1% of the respondents reported that making their children brush themselves was a tiring task.

It was found that parents frequently faced challenging behavior during toothbrushing when their children were between 18 and 24 months old. Those challenging behaviors of children included closing mouths, crying, and wanting to do the toothbrushing by themselves [13]. Similar findings have been reported in the current study, where nearly 33% of mothers reported that they had the most difficulty during PSB when their children were between 1 and 2 years of age. Furthermore, in this study, nearly three quarters of the mothers found it easy and only one quarter found it hard to maintain their children’s oral health care in their daily routine. Similar findings were observed by Gussy et al. [50], where 44% of parents expressed confidence in brushing their children’s teeth. Furthermore, in this study, almost all of the mothers said they had the greatest responsibility for ensuring their children’s oral hygiene, as in the study of Dadalto et al. [24]. 

Dadalto et al. [24] examined the toothbrushing behaviors of children aged 12–38 months at home. Collaborative children who showed co-operative and participatory behaviors comprised 58.4% of the sample. Researchers reported that noncollaborative behavior was more frequent when the mother had lower education and low family income and collaborative behavior was associated with higher maternal education. Similar to previous reports [24,53], in this study, most of the children showed co-operative and participant behaviors rather than resistant, uncooperative, and independent behaviors. Since the dentist mothers who participated in our study had high levels of education, the findings support the assumption of Dadalto et al. [24]. Noncompliant, difficult behaviors were reported as the most common barrier to toothbrushing by 20% of mothers in the current study. In addition, the application of physical restraint to noncooperative children in order to properly brush their teeth was not preferred by most of the mothers in this study, as in previous studies [29,47]. Instead, almost all mothers thought that parents’ ability to manage children’s behavior (parental skills) and interpersonal communication skills are important in protecting children’s oral and dental health. In the study of Huebner and Riedy [18], it was reported that mothers did not want to make their children upset and scared about toothbrushing in the future.

The most common barrier to toothbrushing related with children was falling asleep at home or in the car in the current study. The other barriers were children did not want to brush their teeth at that time, sucking, biting, or chewing the toothbrush during toothbrushing, taking over the toothbrush and wanting to perform brushing on their own, wanting to stop toothbrushing when they got bored, playing with the toothbrush, toothpaste, or water during toothbrushing, and not being co-operative and showing resistance and refusal to open the mouth, respectively. As reported in our study, barriers detected in previous studies included pain during teething [47], tantrums [47], playing with the toothbrush, toothpaste, or water during brushing [76], taking over the toothbrush and wanting to perform the brushing on their own [29], refusal to open their mouth [29], falling asleep at home [13,29], disliking the taste of toothpaste [29], throwing the toothbrush, lying on the ground, and hitting their legs [13]. 

Additionally, there are also some barriers related with mothers in the oral health promotion of their young children. In this study, being tired was the most common barrier reported by dentist mothers. The other barriers were being outside the normal routine, not having enough time in the morning on the way to work, being sick, difficulties in guiding children’s behavior, and difficulties in controlling children, respectively. Twenty-eight percent of mothers stated that they experienced no barriers. Feeling confident and self-efficacy are some of the most important specialties in order to perform dyadic toothbrushing. In accordance with Duijster et al. [47] and Huebner and Riedy [18], nearly all mothers felt confident and had high self-efficacy in order to successfully establish toothbrushing for their children. Parenting skills, including manual skills and behavior management skills, are important to convince children when they do not want to brush [13,17,26,77,78]. In the current study, only 14.8% mothers thought that they had to develop their parenting skills and only one mother felt her hand skills were not sufficient. As reported in our study, barriers also detected in previous studies were not having enough time in the morning on the way to work [18,29,47], stress [13,29], forgetting the toothbrushing of their children [13,29], being outside the normal routine, such as on vacation or at the grandparents’ home [77], and a lack of time [32].

To overcome these mentioned barriers, parents should use facilitators in order to engage the child’s co-operation. In this study, the most common facilitator was brushing teeth together with the children. Being a model for the child during toothbrushing was also reported in the studies of Elison et al. [29], Hubner and Riedy [18], and Duijster et al. [47]. Moreover, 31.8% of all mothers in our study included toothbrushing in the bedtime routine, as mentioned by previous studies [29,79]. Researchers [29] have also reported that initiating toothbrushing as early as possible helps to establish a routine. Daily routinization of toothbrushing was also reported in previous studies [13,18,29,47]. As reported in our study, facilitators mentioned in previous studies included turning toothbrushing into a game [18,29], making sounds like ‘eee, aaa’ [29], performing toothbrushing in order [29], letting the child brush on their own if they do not allow the parent to brush [13,29], rewarding [29], involvement of the father in toothbrushing with the mother [29], explaining the importance of toothbrushing/consequences of not brushing [18], singing a song to the children during toothbrushing [18,47], using a toothbrushing chart/sticker [18,47], choosing toothbrush/toothpaste that the children like, using reminders [18], and praising the child’s toothbrushing [47].

### Strength and Limitations

To the best of our knowledge, this is the first study conducted on dentist mothers who understand the importance of primary teeth and have sufficient knowledge related to oral health. Online questionnaire studies have become one of the main survey methods used by researchers due to their ease of design and the advantages in terms of cost, time, and effort. By this way, it is possible to reach a much larger sample population by crossing geographical boundaries conducted over the internet [80]. Thus, further studies conducted among various countries with larger sample size are encouraged. However, as an online questionnaire, this study has some limitations, such as lack of sufficiently motivating participants to respond, lack of opportunity for some participants to reach the questionnaire, being limited to internet users, and risk of participating without meeting the inclusion criteria. 

This study included detailed information about early childhood caries and contributes by extensively highlighting the most important points about the oral health care of young children. It also encourages dentists to better understand the barriers/challenges faced by parents during parental supervised brushing (PSB) and summarizes the facilitators used in order to improve parental skills, self-efficacy, and achieve better PSB. We believe that this study can serve as a clinical guide for dentists in order to better motivate, educate, and promote preventive behavior shaping of the caregivers of baby/child patients. 

## 5. Conclusions

In conclusion, oral health knowledge is mandatory for the implementation of good oral health behaviors. Without knowledge, there will be no good attitudes and practices towards oral health care. However, reflection of the knowledge in attitudes and practices constitutes a second and important step on the path to achieving good oral health. Although dentist mothers have sufficient knowledge for promoting oral and dental health, they cannot always have ideal attitudes and behaviors and they may encounter various barriers regarding themselves and their children in practical applications. So, this study showed that providing oral care for young children goes beyond ‘knowledge’ and is sometimes ‘challenging’; however, it could be ‘possible’ by developing and implementing the most appropriate solution strategies suitable for each unique family and child.

## Figures and Tables

**Table 1:** Gender and age distribution of participants.

	n	Mean	Median	Minimum	Maximum
Mothers’ age	156	36.12 ± 4.74	35	25	54
Girls’ number and age	79	4.28 ± 2.33	4	0	10
Boys’ number and age	77	4.32 ± 2.31	4	0	9
Total number and age of children	156	4.30 ± 2.31	4	0	10

**Table 2:** Education status of parents.

		n	%
Fathers’ Education Level	General Dentist–Dental specialist	43	27.39
	Master’s–PhD degree	48	30.57
	University	61	38.85
	High School	4	3.18
	Elementary school	0	0
Dental specialties of mothers	General Dentist	80	51.28
	Pedodontics	34	21.79
	Orthodontics	15	9.62
	Restorative Dentistry	5	3.21
	Oral and maxillofacial surgery	1	0.64
	Endodontics	5	3.21
	Periodontics	7	4.49
	Prosthodontics	9	5.77

**Table 3:** Frequency distribution of children’s nutrient consumption.

	Never		Once a Week		Several Times a Week		Once a Day		Many Times during the Day	
	n	%	n	%	n	%	n	%	n	%
Milk products	3	1.91	5	3.18	31	19.75	54	34.39	64	40.76
Fresh vegetables and Fruits	1	0.65	1	0.65	24	15.48	67	43.23	62	40
Carbonated beverages	135	87.10	15	9.68	3	1.94	2	1.29	0	0
Chocolate and cookies	33	21.15	27	17.31	75	48.08	18	11.54	3	1.92
Snacks and potato chips	104	66.67	32	20.51	20	12.82	0	0	0	0
Candy and Sweet foods	91	58.71	36	23.23	27	17.42	1	0.65	0	0

**Table 4:** Frequency distribution of answers to questions related with the night feeding habits of children.

		n	%
Until what age did your child perform night feeding?	Still continues	21	13.38
	3 to 6 months old	9	5.73
	6 months–1 year old	16	10.19
	1 year old	22	14.01
	1 to 2 years old	61	38.85
	2.5 years old	20	12.74
	2.5–3 years old	4	2.55
	3–4 years old	3	1.91
	4+ years old	1	0.64
Did your child ever consume formula milk during the night?	Always	9	5.77
	Generally	25	16.03
	Sometimes	26	16.67
	Never	96	61.54
Did your child ever consume breast milk during the night?	Always	68	43.31
	Generally	46	29.30
	Sometimes	27	17.20
	Never	16	10.19
Which oral hygiene aid did you use to clean your child’s teeth after night feeding?	I could not clean after night feeding	114	74.03
	Teeth cleaning wipes	3	1.95
	Wet gauze	20	12.99
	Finger brush—without paste	9	5.84
	Finger brush and fluoride-free baby/child paste	1	0.65
	Toothbrush—without paste	5	3.25
	Toothbrush and fluoride-free baby/child paste	1	0.65
	Toothbrush and fluoridated baby/child paste	1	0.65
If you could not, what was your reason for not being able to clean your child’s teeth after night feeding?	Not to wake up the baby	102	69.86
	Being tired	5	3.42
	Do not care about dental caries in primary teeth	1	0.68
	Any other reasons	38	26.03

**Table 5:** Frequency distribution of answers to questions related with the oral health status/habits of children.

		n	%
When did you start cleaning your child’s teeth?	Immediately after he/she was born, I cleaned the tooth crests and continued since then	45	28.66
	After the eruption of the first primary teeth	73	46.50
	After the age of 1 year	25	15.92
	After the age of 2 years	12	7.64
	I have not brushed them yet	2	1.27
What is the duration of toothbrushing?	Less than 2–3 min	99	64.29
	2–3 min	52	33.77
	More than 3 min	3	1.95
Which oral hygiene aids did you use to clean your child’s teeth?	I haven’t cleaned them yet	1	0.65
	Wet gauze	1	0.65
	Teeth cleaning wipes	0	0
	Finger brush—without paste	10	6.45
	Finger brush and fluoride-free baby/child paste	1	0.65
	Finger brush and fluoridated baby/child paste	1	0.65
	Toothbrush—without paste	29	18.71
	Toothbrush and fluoride-free baby/child paste	55	35.48
	Toothbrush and fluoridated baby/child paste	57	36.77
How often and when are your child’s teeth brushed?	After each feeding	10	6.45
	2 times a day—morning and evening before bedtime	65	41.94
	Once a day—just in the morning	7	4.52
	Once a day—Just in the evening before bedtime	60	38.71
	3 times a week	13	8.39
	Rarely/never	0	0
What is the fluoride concentration of your child’s toothpaste?	Fluoride Free	74	50.00
	<600 ppm	29	19.59
	1000 ppm	12	8.11
	1000–1500 ppm	9	6.08
	I do not know	24	16.22
What amount of toothpaste do you use for your child while toothbrushing?	Smear Layer	77	56.62
	Pea size	59	43.38
Are you satisfied with your child’s toothbrushing frequency?	Always	34	21.94
	Generally	78	50.32
	Sometimes	33	21.29
	Never	10	6.45
How often is your child’s tongue surface brushed?	Always	20	12.82
	Generally	24	15.38
	Sometimes	60	38.46
	Never	52	33.33
Does your child accept you as an example for toothbrushing?	No	5	3.18
	Yes	152	96.82
Do you think that you are an example to your child for toothbrushing?	No	7	4.46
	Yes	150	95.54
Could your child perform toothbrushing at school?	No	109	78.99
	Yes	29	21.01
Should a toothbrushing protocol be prepared in schools under the supervision of teachers?	No	31	20.81
	Yes	118	79.19
How would you describe your child’s oral health?	Poor	2	1.28
	Average	15	9.62
	Good	87	55.77
	Excellent	52	33.33
How would you describe your own oral health?	Poor	13	8.28
	Average	114	72.61
	Good	30	19.11
	Excellent	88	58.67
How often do you lift your child’s lip and check for opaque enamel lesions, brown or black spotting?	Always	82	52.90
	Generally	38	24.52
	Sometimes	33	21.29
	Never	2	1.29
Have you ever given a cup, fork, spoon, pacifier, or bottle to your child after you used it?	Always	2	1.28
	Generally	12	7.69
	Sometimes	55	35.26
	Never	87	55.77
Does your child have white spot lesions?	No	138	87.90
	Yes	19	12.10
Did you apply fluoride varnish/gel to your child?	No	112	71.79
	Yes	44	28.21
Does your child have cavitated carious lesions?	No	130	83.33
	Yes	26	16.67
Did your child suffer from dental trauma to his/her anterior teeth?	No	137	87.82
	Yes	19	12.18

**Table 6:** Comparative distribution of answers to some questions according to the age groups of children.

		0–2 Years Old		2–6 Years Old		>6 Years Old		
		n	%	n	%	n	%	*p*
Which oral hygiene aids did you use to clean your child’s teeth?	I have not clean them yet	1	5.56%	0	0.00%	0	0.00%	0.020
	Wet gauze	0	0.00%	0	0.00%	1	2.78%	0.192
	Finger brush-without paste	6	33.33%	2	2.00%	2	5.56%	0.001
	Finger brush and fluoride-free baby/child paste	0	0.00%	1	1.00%	0	0.00%	0.762
	Finger brush and fluoridated baby/child paste	0	0.00%	1	1.00%	0	0.00%	0.762
	Toothbrush—without paste	8	44.44%	15	15.00%	5	13.89%	0.008
	Toothbrush and fluoride-free baby/child paste	3	16.67%	43	43.00%	9	25.00%	0.030
	Toothbrush and fluoridated baby/child paste	0	0.00%	38	38.00%	19	52.78%	0.007
What is the fluoride concentration of your child’s toothpaste?	Fluoride Free	16	94.12%	46	48.42%	12	34.29%	0.001
	<600 ppm	1	5.88%	22	23.16%	6	17.14%	
	1000 ppm	0	0.00%	7	7.37%	5	14.29%	
	1000–1500 ppm	0	0.00%	3	3.16%	6	17.14%	
	I don’t know	0	0.00%	17	17.89%	6	17.14%	
What amount of toothpaste do you use for your child while toothbrushing?	Smear layer	9	69.23%	53	59.55%	15	44.12%	0.190
	Pea size	4	30.77%	36	40.45%	19	55.88%	
Did you apply flüoride varnish/gel to your child?	No	18	100.00%	81	80.20%	12	33.33%	0.0001
	Yes	0	0.00%	20	19.80%	24	66.67%	
How long did your child use a pacifier?	Never used	9	50.00%	44	43.56%	17	47.22%	0.335
	Currently using	6	33.33%	3	2.97%	0	0.00%	0.0001
	3 to 6 months of age	2	11.11%	7	6.93%	0	0.00%	0.185
	6 months to 1 year of age	0	0.00%	3	2.97%	3	0.00%	0.237
	1 year of age	0	0.00%	4	3.96%	1	8.33%	0.798
	1 to 2 years of age	1	5.56%	20	19.80%	7	2.78%	0.340
	2.5 years of age	0	0.00%	14	13.86%	8	19.44%	0.086
	2.5 to 3 years of age	0	0.00%	4	3.96%	0	22.22%	0.334
	3 to 4 years of age	0	0.00%	1	0.99%	0	0.00%	0.764
	4 and more years of age	0	0.00%	1	0.99%	0	0.00%	0.764
In ensuring your child’s oral hygiene	I brush my childs teeth	14	82.35%	35	34.65%	4	11.11%	0.0001
	I supervise my child during toothbrushing and I often perform the brushing	2	11.76%	42	41.58%	9	25.00%	0.022
	I supervise my child during toothbrushing and sometimes I brush	0	0.00%	21	20.79%	16	44.44%	0.008
	I only supervise my child during toothbrushing and I do not brush	1	5.88%	3	2.97%	6	16.67%	0.016
	I do not supervise my child during toothbrushing; I only remind and give advice.	0	0.00%	0	0.00%	1	2.78%	0.192

**Table 7:** Frequency distribution of answers to questions related with the bad oral habits of children.

		n	%
How long did your child use a pacifier?	Never used	71	45.51
	Currently using	9	5.77
	3 to 6 months of age	9	5.77
	6 months to 1 year of age	6	3.85
	1 year of age	5	3.21
	1 to 2 years of age	28	17.95
	2.5 years of age	22	14.10
	2.5 to 3 years of age	4	2.56
	3 to 4 years of age	1	0.64
	4 and more years of age	1	0.64
Did your child ever use a pacifier during night sleep?	Yes, I took it out of his/her mouth after they feel asleep.	45	29.03
	Yes, he/she needed a pacifier all night long.	23	14.84
	No	87	56.13
Did your child ever suck his/her finger? If yes, until what age did she/he suck?	Never sucked	142	91.61
	Currently sucking	1	0.65
	3 to 6 months of age	6	3.87
	6 months to 1 year of age	1	0.65
	1 year of age	1	0.65
	1 to 2 years of age	3	1.94
	2.5 years of age	0	0
	2.5 to 3 years of age	1	0.65
	3 to 4 years of age	0	0
	4 and more years of age	0	0
Did your child ever perform mouth breathing?	No	115	73.25
	Yes	42	26.75
Did your child perform tongue-thrusting?	No	151	96.79
	Yes	5	3.21
Did your child ever perform lip-sucking?	No	151	97.42
	Yes	4	2.58
Did your child ever perform lip biting?	No	146	94.19
	Yes	9	5.81
Did your child ever perform nail biting?	No	141	90.97
	Yes	14	9.03
Did your child ever have bruxism?	No	115	74.19
	Yes	40	25.81
Has your child developed any malocclusion (anterior open bite, increased overjet, narrow maxilla, or unilateral crossbite) due to bad oral habits?	No	140	89.74
	Yes	16	10.26

**Table 8:** Frequency distribution of answers to questions related with parental supervised toothbrushing.

		n	%
Until what age do you think that you should brush your child’s teeth?	1–2	3	1.91
	3–4	31	19.75
	5–6	44	28.03
	7–8	79	50.32
In ensuring your child’s oral hygiene	I brush my child’s teeth.	53	34.19
	I supervise my child during toothbrushing and I often perform the brushing	54	34.84
	I supervise my child during toothbrushing and sometimes I brush.	37	23.87
	I only supervise my child during toothbrushing and I do not brush	10	6.45
	I do not supervise my child during toothbrushing; I only remind and give advice.	1	0.65
Is it easy to perform the oral health care of your child in your daily routine?	Very easy	21	13.46
	Easy	97	62.18
	Hard	32	20.51
	Very hard	6	3.85
Do you feel that you have the greatest responsibility for ensuring your child’s oral hygiene?	Always	87	55.77
	Generally	62	39.74
	Sometimes	6	3.85
	Never	1	0.64
How is your child’s behavior during toothbrushing?	Co-operative—The child allows me to brush her/his teeth.	32	20.65
	Participant—The child brushes her/his teeth before or after me. After the child allows me to brush her/his teeth	88	56.77
	Resistant—The child allows me to partially brush his/her teeth.	24	15.48
	Uncooperative—The child brushes with physical restraint	4	2.58
	İndependent—The child does not allow me to perform toothbrushing. The child brushes his/her teeth on their own	7	4.52
If your child has shown resistance to toothbrushing before, have you ever done brushing by applying physical restraint?	Always	5	3.25
	Generally	6	3.90
	Sometimes	34	22.08
	Never	109	70.78
Have you ever forced your child to brush their teeth?	No	70	44.87
	Yes	86	55.13
Please mark the age range (s) that you experienced the most difficulties while performing oral cleaning of your child	0–3 months of age	9	5.26
	3–6 months of age	12	7.01
	6 months to 1 year of age	31	18.12
	1 to 2 years of age	56	32.74
	2 to 3 years of age	40	23.39
	3 to 5 years of age	13	7.60
	5 to 8 years of age	10	5.84
Do you think that parents’ ability to manage their children’s behavior (parental skills) and interpersonal communication skills are important in protecting children’s oral and dental health?	Yes	152	99.35
	No	0	0
	I do not know	1	0.65

## Data Availability

The data that support the findings of this study are available on request from the corresponding author. The data are not publicly available due to privacy and ethical restrictions.
